# Protective Effect of *Laminaria japonica* with Probiotics on Murine Colitis

**DOI:** 10.1155/2014/417814

**Published:** 2014-05-18

**Authors:** Seok-Jae Ko, Youngmin Bu, Jinhyun Bae, Yu-mi Bang, Jinsung Kim, Hyejung Lee, Lee Beom-Joon, Yoo Hye Hyun, Jae-Woo Park

**Affiliations:** ^1^College of Korean Medicine, Kyung Hee University, 26 Kyungheedae-ro, Dongdaemun-gu, Seoul 130-701, Republic of Korea; ^2^Acupuncture and Meridian Science Research Center, College of Korean Medicine, Kyung Hee University, 26 Kyungheedae-ro, Dongdaemun-gu, Seoul 130-701, Republic of Korea; ^3^Department of Pharmacy, College of Pharmacy, Hanyang University, Ansan 426-791, Republic of Korea

## Abstract

Inflammatory bowel disease (IBD) is a chronically relapsing inflammatory disorder of the gastrointestinal tract. Most IBD treatments are unsatisfactory; therefore, various dietary supplements have emerged as promising interventions. *Laminaria japonica* (LJ) is an edible seaweed used to regulate digestive symptoms. Probiotics have been reported to improve digestive problems and their simultaneous administration with seaweeds has been shown to produce synergistic therapeutic effects. Here, we investigated the effect of LJ combination with probiotics on dextran sodium sulfate-induced colitis model in mice. Aqueous LJ extracts (LJE) at doses from 100 to 300 mg/kg and probiotics at a dose of 300 mg/kg were orally administered for 7 days. Body weight, colon length, histological score, macroscopic damage, and the levels of cytokines IFN-**γ**, IL-1**β**, IL-6, IL-10, IL-12 (P40), IL-12 (P70), IL-17, and TNF-**α** were assessed. LJE alone caused a significant improvement of colitis signs such as colon length, histological score, and IL-1**β** and IL-6 production. LJE and probiotics demonstrated a synergistic effect by the histological score and levels of IL-1**β**, IL-6, and IL-12 (P40) but not IFN-**γ**, IL-10, and IL-12 (P70). In conclusion, LJE was effective in inducing protection against colitis in mice and acted synergistically with probiotics.

## 1. Introduction

Inflammatory bowel disease (IBD) is a group of inflammatory conditions of gastrointestinal tract with two major types including ulcerative colitis (UC) and Crohn's disease (CD). IBD is considered an autoimmune disease and the pathogenesis depends on complex interactions between genetic and environmental factors and innate and adaptive immune mechanisms; the exact cause of IBD is still unknown [1]. Current IBD treatments include aminosalicylates, corticosteroids, inhibitors of tumor necrosis factor alpha (TNF-*α*), antibiotics, and immunosuppressants [2]. However, these agents have poor tolerability and insufficient therapeutic efficacy; therefore, the need for alternative therapeutic approaches is increasing [3, 4].

Seaweed* Laminaria japonica *(LJ) has been used as a herbal medicine for the treatment of gastrointestinal problems such as vomiting and hemorrhoids in Korea [5]. LJ contains various beneficial ingredients, including alginate and fucoidans known for their dietary benefits and preventive effects on constipation [6] and inflammation [7]. The effects of seaweeds on IBD have been previously shown with* Sargassum pallidum* [8] and* Samiunkyuntang *(a herbal medicine composed of seaweeds* Sargassum pallidum *and* Zostera marina*) [9]. However, the effect of LJ on colitis has not been investigated.

Recent research on new functional food composed of LJ and LAB has shown that the combination exhibits high antioxidant activity and protective effects against liver damage, obesity, hypertension, stress, and insomnia [10, 11]. The lactic acid bacteria (LAB), usually found in decomposing plants and dairy products, comprise a group of Gram-positive, acid-tolerant rods or cocci that exhibit common metabolic and physiological characteristics. Many experimental reports have demonstrated that LAB can improve functions of indigenous microflora and have positive effects on IBD [12–14]. The simultaneous administration of herbal medicines and probiotics containing LAB as a main component has become a popular treatment for many abdominal symptoms in Korea [15]. We have previously shown that the combination of herbal extracts and probiotics may produce a positive synergetic effect in treatment of gastrointestinal diseases [16]. Duolac7S (DUO) is a probiotic mixture containing seven LAB species including* Bifidobacterium*,* Lactobacillus*, and* Streptococcus*. They are easily available, do not require a prescription, and are extensively used to relieve abdominal symptoms [17] by restoring the ecological balance in intestinal microflora [18]. However, in IBD the effect of DUO and its combination with LJ has not been investigated. Here, we applied LJ in a dextran sodium sulfate- (DSS-) induced colitis model in mice and assessed body weight, colon length, and histological damage. Mechanistic studies were conducted by profiling cytokine expression by using the biometric multiplex signaling method. Cytokines including IL-6 and TNF-*α*, which play a crucial role in acute DSS-induced colitis model [19], were analyzed. We also treated mice with a combination of LJ and DUO in order to investigate a possible synergetic effect on colitis.

## 2. Materials and Methods

### 2.1. Animals

Balb/c mice (21-22 g) were obtained from Taconic Korea (Daehan Bio Link, Seoul, Korea). Animals were housed at an ambient temperature of 21°C and 46 ± 10% humidity and allowed food and water* ad libitum*. All mice were acclimated for a week before the experiment. All experimental procedures were conformed to the international guidelines “Principles of Laboratory Animals Care” (NIH publication number 85-23, revised 1985, and Kyung Hee University 2006). The international animal ethical committee of Kyung Hee University approved the experimental protocol (KHUASP (SE)-09-036).

### 2.2. Preparation of Herbal Water Extract and Probiotics

Dried stem bark of LJ was purchased from Kyung Hee Hanyak Co. (Seoul, Republic of Korea). A sample of 100 g was boiled in water for 2.5 h, filtered, and freeze-dried to a powdered form (code name: PJW-042, yield = 18.01%). The voucher specimen of LJ extract (LJE) was deposited in the herbal laboratory of College of Korean Medicine, Kyung Hee University. LJE was used as a solution in distilled water.

DUO (purchased from Cell Biotech Co., Ltd., Gimpo, Korea) is a probiotic mixture containing multiple viable species of three bacterial genera: three strains of* Bifidobacterium* (*B. brevis*,* B. lactis*, and* B. longum*), three strains of* Lactobacillus* (*L. acidophilus*,* L. plantarum*, and* L. rhamnosus*), and one strain of* Streptococcus* (*S. thermophilus*). Each DUO capsule contains 5 × 10^9^ bacteria (approximately 7 × 10^8^ bacteria for each strain).

To investigate the therapeutic effects of LJE or LJE combination with DUO (LJE + DUO), mice were randomly allocated to seven groups (*n* = 7 per group) and treated with DSS only (control), LJE (100 and 300 mg/kg), DUO (300 mg/kg), and LJE (100 and 300 mg/kg) + DUO (300 mg/kg) or left untreated (normal).

### 2.3. Induction of Colitis

Male Balb/c mice (7 weeks old) were provided with drinking water containing 5% DSS (USB Corporation, Cleveland, OH, USA)* ad libitum* for 7 days. Treatment groups received LJE, DUO, or LJE + DUO administered twice a day by feeding tube for the duration of the experiment. The animals were sacrificed on day 7 and subjected to pathological analysis.

### 2.4. Assessment of Mouse Weight and Colon Length

Mouse weight was measured daily from day 0 to day 7 at 9:30 am every day. The colon was isolated immediately after the last weight check. Colon length was measured from the cecum to the anus using a vernier caliper (Mitutoyo, Otopeni, Romania).

### 2.5. Clinical Parameters

The clinical score was measured by the modified method used in the previous study [20]. Two investigators blinded to the protocol daily assessed the clinical score of DSS treatment. Spontaneous behavior and posture were scored as 4, moving (+++) without hunching; 3, moving (++) without hunching; 2, moving (+) with hunching; 1, moving (±) with hunching; 0, moving (−) with hunching., Coat and piloerection were evaluated as 4, normal state; 3, clean and yellowish (+) without piloerection; 2, yellowish (+) with piloerection (+); 1, dirty and yellowish (+++) with piloerection (++); and 0, yellowish (light brown) with piloerection (+++). Cleaning of perianal region was scored as follows: 4, normal state; 3, with stool (+) trace; 2, with stool (++) and blood (+) trace; 1, with stool (+++) and blood (++) trace; and 0, herniation with blood (+++). The final clinical score was obtained by summarizing all the scores.

### 2.6. Macroscopic Pathology

The macroscopic score was measured by the modified method of the previous study [21]. Two investigators blinded to the protocol assessed the score at day 7 after DSS treatment. Edema and colon sickness were scored as follows: 4, no edema, colon thickness 0.1-0.2 mm; 3, edema (±), 0.2–0.25 mm; 2, edema (+), 0.25–0.30 mm; 1, edema (++), 0.3–0.35 mm; and 0, edema (+++) > 0.35. Overall health was scored as follows: 4, no bleeding with normal stool; 3, no bleeding with semiformed stool; 2, fecal blood (+) with pasty and semiformed stool; 1, fecal blood (++) with tar stool (+); and 0, bleeding (+++) with tar stool (++). The macroscopic score was obtained by summarizing all scores.

### 2.7. Histology

Colons were fixed in 10% buffered formalin and embedded in paraffin. Histological sections cut from the paraffin blocks were stained with hematoxylin and eosin. In a blind fashion, the scoring of histological damage was divided into two categories: inflammatory cell infiltration and ulceration. Inflammatory cell infiltration was assessed in each layer of the colon, including surface epithelium, cryptal glands, stroma, submucosa, and transmural layer and graded on a scale from 0 to 3 (0, none; 1, mild; 2, moderate; and 3, severe). The severity of ulceration was graded histologically on a scale from 0 to 4 (0, none; 1, mild and focal surface; 2, mucosal layer; 3, submucosal layer; and 4, transmural layer) [22, 23]. We modified and established a 0–19 scoring system by summarizing all the scores.

### 2.8. Collection of Colonic Mucosa and Biometric Multiplex Cytokine Profiling

Isolated colons were snap-frozen and stored at −70°C. The mucosa was scraped from the muscle layer of the colon and weighed using analytical balance (Ohaus Voyager, Parsippany, NJ, USA). A sample of 100 ± 10 mg was dissolved in triple-detergent lysis buffer (50 mM Tris-HCl, pH 8.0, 150 mM NaCl, 0.1% SDS, 1% NP-40, 0.02% sodium azide, 0.5% sodium deoxycholate, and 1 mM phenylmethylsulfonyl fluoride) and homogenized. The levels of eight cytokines involved in a broad spectrum of immune and inflammatory mechanisms were measured in parallel following the induction of colitis [19].

Interferon (IFN)-*γ*, IL-1*β*, IL-6, IL-10, IL-12 (P40), IL-12 (P70), IL-17, and TNF-*α* in the mucosa samples were analyzed using the biometric multiplex cytokine assay (Millipore, Billerica, MA, USA) according to the manufacturer's protocol.

### 2.9. Statistical Analysis

All results were expressed as mean values with standard errors. Data were analyzed by one-way ANOVA followed by Dunnett's test to compare treatment groups with the control group. *P* < 0.05 was regarded as statistically significant.

## 3. Results

### 3.1. Body Weight and Colon Length

**Figure 1:**
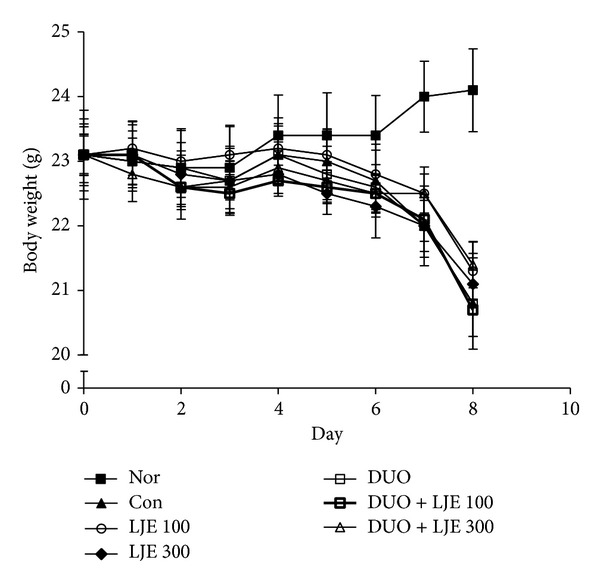
Effects of LJE or DUO on mice weight in DSS-induced colitis. Data are expressed as mean ± S.E.M (*n* = 7). Nor: normal mice without any treatment, Con: mice provided with only 5% DSS treatment, LJE: the water extract of* Laminaria japonica*, DUO: Duolac7S, LJE + DUO: cotreatment of LJE and DUO, 100: the dosage of LJE 100 mg/kg, and 300: the dosage of LJE 300 mg/kg.

From day 5, the weight of mice in all groups except normal group has shown a decreasing tendency. Compared with the control group, LJE, DUO, and LJE + DUO groups showed no significant protective effect against weight loss induced by DSS (Figure 1).

**Figure 2:**
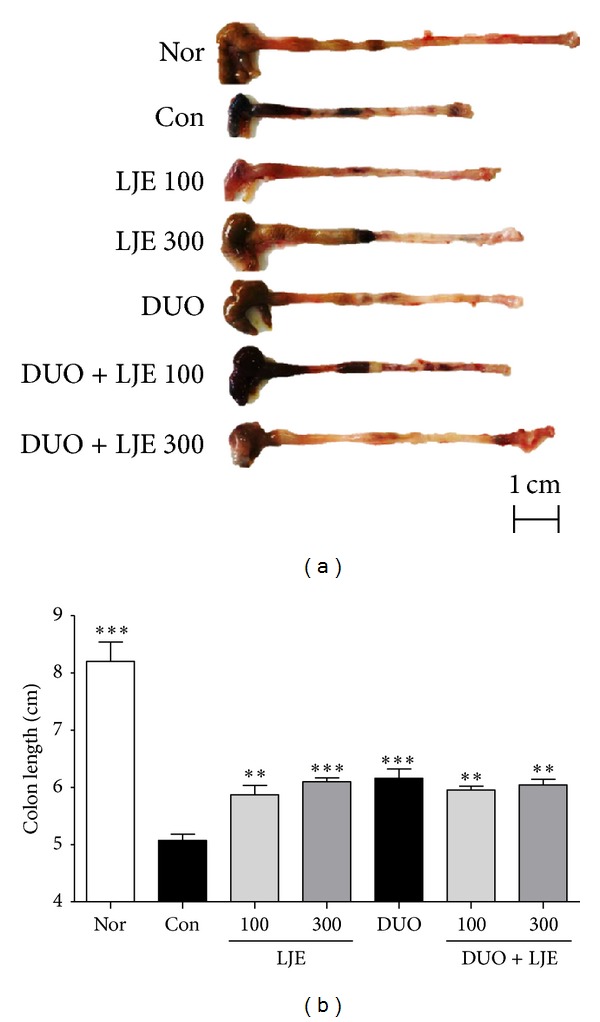
Effects of LJE or DUO on colon length in DSS-induced colitis. (a) The representative colon photos at 7 days on DSS-induced colitis, (b) a graph showing the data of colon length. Data are expressed as mean ± S.E.M (*n* = 7). Nor: normal mice without any treatment, Con: mice provided with only 5% DSS treatment, LJE: the water extract of* Laminaria japonica*, DUO: Duolac7S, LJE + DUO: cotreatment of LJE and DUO, 100: the dosage of LJE 100 mg/kg, and 300: the dosage of LJE 300 mg/kg; **P < 0.01, ***P < 0.001 versus control group analyzed by one-way ANOVA with Dunnett's post hoc test.

However, DSS-dependent colon shortening was significantly inhibited in mice treated with LJE (100 and 300 mg/kg), DUO, DUO + LJE (100 mg/kg), and DUO + LJE (300 mg/kg) (*P* < 0.01, *P* < 0.001, *P* < 0.001, *P* < 0.01, and *P* < 0.01, resp.) (Figures 2(a) and 2(b)).

### 3.2. Clinical Findings

**Figure 3:**
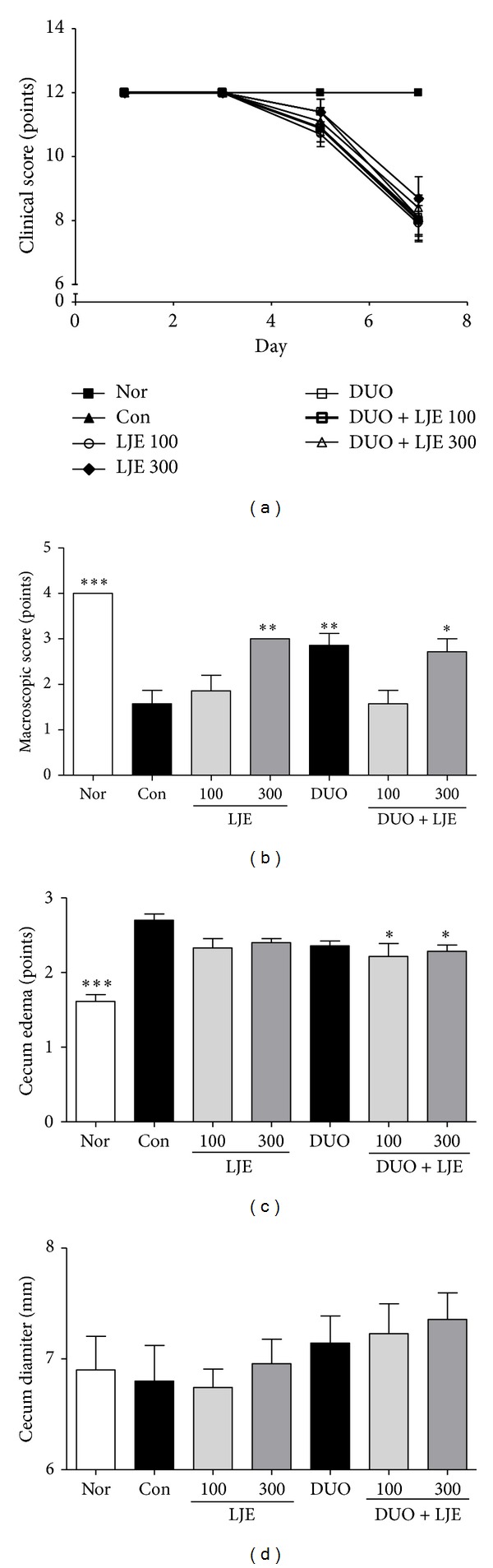
(a) Effects of LJE or DUO on clinical score in DSS-induced colitis. (b) Effects of LJE or DUO on gross macroscopic finding of colon in DSS-induced colitis. (c) Effects of LJE or DUO on cecum edema in DSS-induced colitis. (d) Effects of LJE or DUO on cecum diameter in DSS-induced colitis. Data are expressed as mean ± S.E.M (*n* = 7). Nor: normal mice without any treatment, Con: mice provided with only 5% DSS treatment, LJE: the water extract of* Laminaria japonica*, DUO: Duolac7S, LJE + DUO: cotreatment of LJE and DUO, 100: the dosage of LJE 100 mg/kg, and 300: the dosage of LJE 300 mg/kg; *P < 0.05, **P < 0.01, and ***P < 0.001 versus control group analyzed by one-way ANOVA with Dunnett's post hoc test.

Animals from all groups appeared healthy and active, with relatively clean perianal lesions until day 5, when clinical parameters started to worsen. As shown in Figure 3(a), the treatment with LJE or DUO + LJE caused no significant improvement of the clinical score.

### 3.3. Macroscopic Findings

Control animals developed clinical signs of colitis such as severe edema of the colon, fecal blood, or tar stool. On the other hand, the cecal diameter in control DSS-treated group was not significantly different to that in healthy animals.

The treatment with LJE (300 mg/kg), DUO, and DUO + LJE (300 mg/kg) showed a significant effect compared to other groups (*P* < 0.01, *P* < 0.01, and *P* < 0.05, resp.) as revealed by the macroscopic score (Figure 3(b)). In addition, DUO + LJE-treated mice showed an improvement of the cecal edema compared to any other groups (*P* < 0.05 for both DUO + LJE groups; Figure 3(c)). However, none of the treatments had significant beneficial effects on the cecum diameter (Figure 3(d)).

### 3.4. Histological Findings

**Figure 4:**
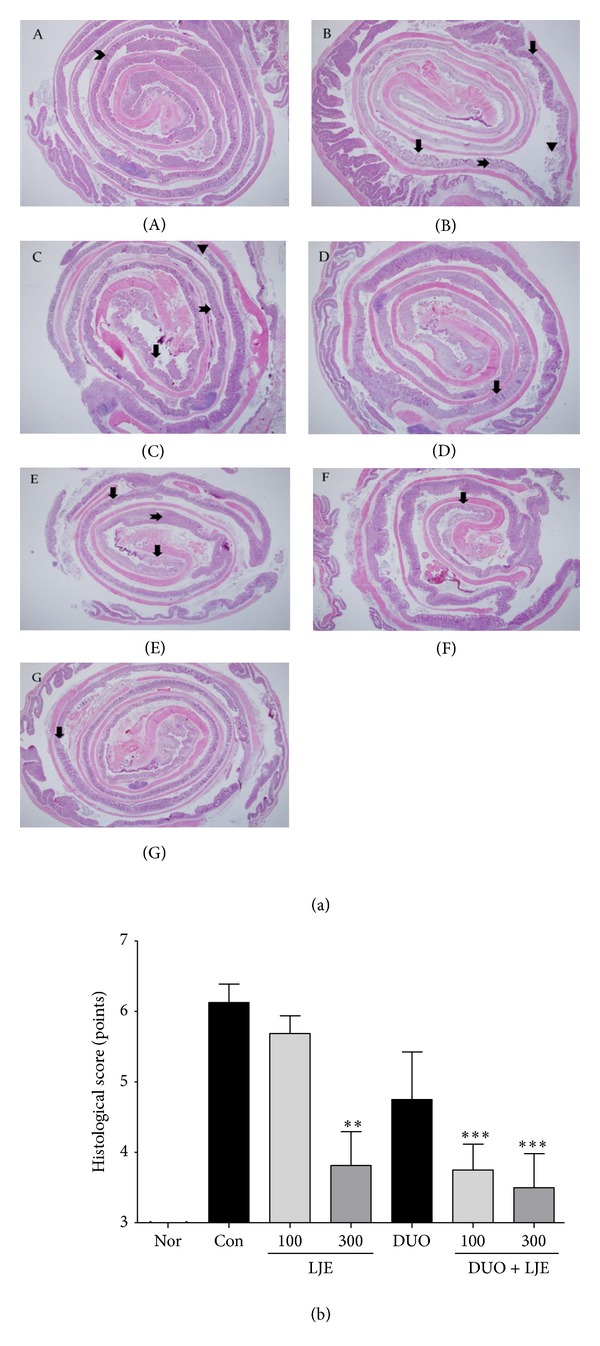
(a) Effects of LJE or DUO on histological findings in DSS-induced colitis. (a)-(A): normal, (a)-(B): control, (a)-(C): LJE 100, (a)-(D): LJE 300, (a)-(E): DUO, (a)-(F): DUO + LJE 100, (a)-(G): DUO + LJE 300, chevron: normal crypt, down arrow: cryptal damage, notches right arrow: infiltration of inflammatory cell, and reversed triangle: ulcer. The whole colon tissues were stained by (H) and (E). The center was the distal part and the boundary was the proximal part of colon (×20). (b) Effects of LJE or DUO on histological scores in DSS-induced colitis. Nor: normal mice without any treatment, Con: mice provided with only 5% DSS treatment, LJE: the water extract of* Laminaria japonica*, DUO: Duolac7S, LJE + DUO: cotreatment of LJE and DUO, 100: the dosage of LJE 100 mg/kg, and 300: the dosage of LJE 300 mg/kg; **P < 0.01, ***P < 0.001 versus control group analyzed by one-way ANOVA with Dunnett's post hoc test.

While the surface epithelium, cryptal glands, mucosa, and submucosa in the normal mice were intact (Figure 4(a)-(A)), DSS-treated animals showed severe damage of the surface epithelium, infiltration of the inflammatory cells, and disruption of the cryptal glands (Figure 4(a)-(B)). The pathology of the colonic mucosal layer was improved by the treatment with LJE, DUO, and LJE + DUO, indicating a protective effect against DSS-induced colitis.

The animals administered LJE (100 mg/kg) showed relatively intact surface epithelium, but cryptal glands at the distal colon were disrupted and ulceration similar to the control group was observed (Figure 4(a)-(C)). However, the increase of LJE concentration to 300 mg/kg showed more protective effects and improved DSS-induced pathology. Though infiltrated inflammatory cells and cryptal damage were observed in several mucosal areas, the overall structure of mucosa and submucosa was relatively better preserved (Figure 4(a)-(D)). The DUO-treated group showed more severe damage of cryptal glands and more ulcers compared to the animals treated with LJE (300 mg/kg) (Figure 4(a)-(E)). Both groups treated with the combination of DUO and LJE showed an improvement in the structure of the surface epithelium and cryptal glands compared to the other groups (Figure 4(a)-(F)); it was especially evident for DUO + LJE (300 mg/kg)-treated mice (Figure 4(a)-(G)).

Regarding the histological score, LJE (300 mg/kg) and DUO + LJE (100 and 300 mg/kg) showed significant protection against histological damage caused by DSS administration (*P* < 0.01, *P* < 0.001, and *P* < 0.001, resp.; Figure 4(b)). LJE treatment demonstrated a dose-dependent protection against histological damage and the combined administration of DUO + LJE (300 mg/kg) caused the maximal improvement in histology.

### 3.5. Changes on Cytokine Levels in Colonic Mucosa

**Figure 5:**
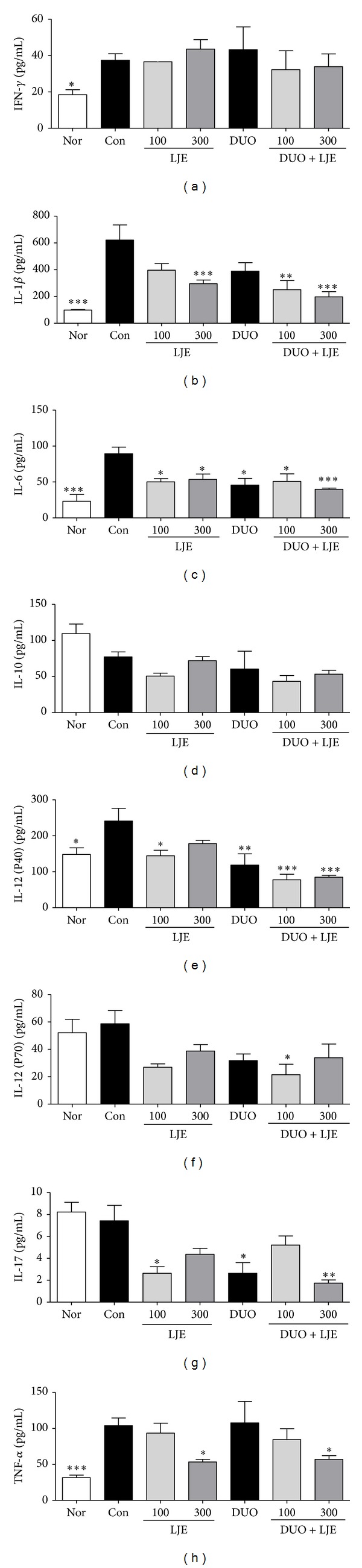
Effects of LJE or DUO on cytokine production in DSS-induced colitis. Data are expressed as mean ± S.E.M (*n* = 7). Nor: normal mice without any treatment, Con: mice provided with only 5% DSS treatment, LJE: the water extract of* Laminaria japonica*, DUO: Duolac7S, LJE + DUO: cotreatment of LJE and DUO, 100: the dosage of LJE 100 mg/kg, and 300: the dosage of LJE 300 mg/kg; *P < 0.05, **P < 0.01, and ***P < 0.001 versus control group analyzed by one-way ANOVA with Dunnett's post hoc test. IFN-*γ*: interferon-*γ* and TNF-*α*: tumor necrosis factor-*α*.

To analyze the influence of LJE and DUO on cytokine production in colonic mucosa during colitis, we measured the levels of representative cytokines by multiplex profiling after 7 days of DSS administration (Figure 5). IFN-*γ* level was markedly increased in the DSS-treated group, and the treatments did not cause any significant changes. However, the level of IL-1*β* in mice administered LJE (300 mg/kg) or combinations of DUO + LJE (100 and 300 mg/kg) significantly decreased compared to that in the control group (*P* < 0.001,  *P* < 0.001, and *P* < 0.001, resp.); there was no significant difference with control in the other groups. IL-6 level in the groups treated with LJE (100 and 300 mg/kg), DUO, and DUO + LJE (100 and 300 mg/kg) significantly decreased compared to that of the control group (*P* < 0.05,  *P* < 0.05,  *P* < 0.05,  *P* < 0.05, and *P* < 0.001, resp.). All treatment groups showed a statistically significant decrease in IL-6, which was more pronounced in animals treated with DUO + LJE (300 mg/kg). The level of IL-10 was not different among the groups. IL-12 (P40) level in animals treated with LJE (100 mg/kg), DUO, or DUO + LJE (100 and 300 mg/kg) significantly decreased compared to that in the DSS-treated control group (*P* < 0.05,  *P* < 0.01,  *P* < 0.001, and *P* < 0.001, resp.); the combination treatment had a stronger effect. However, the treatment with LJE (300 mg/kg) did not change IL-12 levels. The level of IL-12 (P70) in mice treated with DUO + LJE (100 mg/kg) significantly decreased compared to that in the control group (*P* < 0.05); however, no changes were observed in the other treatment groups compared to control. IL-17 levels in the LJE (100 mg/kg), DUO, and DUO + LJE (300 mg/kg) groups significantly decreased compared to that of the control group (*P* < 0.05,  *P* < 0.05, and *P* < 0.001, resp.). DUO + LJE (300 mg/kg) showed the strongest effect, while LJE (300 mg/kg) and DUO + LJE (100 mg/kg) did not cause any changes in IL-17 compared to the DSS group. The level of TNF-*α* in mice treated with LJE (300 mg/kg) and DUO + LJE (300 mg/kg) significantly decreased compared to that of the control group (*P* < 0.05 and *P* < 0.05, resp.), while LJE (100 mg/kg), DUO, and DUO + LJE (100 mg/kg) had no effect.

## 4. Discussion

In the present study, oral administration of LJE at doses 100 and 300 mg/kg and DUO at 300 mg/kg prevented colon shortening, histological damage and induction of proinflammatory cytokines associated with DSS-induced colitis. The effect of the cotreatment with LJE and DUO showed better protective effect against histological damage and induction of cytokines including IL-1*β*, IL-6, and IL-12 (P40) compared to the treatment with LJE or DUO alone.

Inflammatory bowel disease (IBD), which comprises Crohn's disease (CD) and ulcerative colitis (UC), is a chronic autoimmune gastrointestinal condition with uncertain etiology. A recent systematic review reported the increase in the worldwide incidence and prevalence of UC and CD, indicating the emergence of IBD as a global disease [24]. The familial aggregation rate is lower in East Asia but higher in West and South Asia; the genetic susceptibility to IBD in Asian population is different from that in Western countries, where it is not associated with NOD2/CARD15 mutations [25]. IBD etiology remains unknown and is thought to be a result of the complex interaction between genetics and environmental factors; some of them are related to intestinal microflora and innate and adaptive immunity [26]. The mucosal immune system exhibits a substantial homeostatic and inflammation-restraining role by secreting and activating various cytokine mediators [27].

In our model of colitis, the treatment with LJE and DUO inhibited the shortening of the colon; it was particularly evident in animals administered LJE (300 mg/kg), DUO, or DUO + LJE (300 mg/kg). Intestinal bacteria produce inflammatory cytokines including IL-6 and TNF-*α*, which leads to the inflammation of the colonic mucosa, erosion, ulceration, and shortening of the colon [28, 29]. Colon shortening was inhibited by the treatment with LJE or DUO, which also prevented the loss of cryptal glands and epithelial damage. Colon length is considered as a hallmark of experimental colitis, and the histological findings of this study demonstrate that the colon length correlates with the severity of structural damage and the number of infiltrated inflammatory cells. The levels of IL-6 and TNF-*α* production showed the trend similar to that of the colon length, that is, the highest effect in mice treated with LJE (300 mg/kg) and DUO + LJE (300 mg/kg).

The histological findings demonstrate that the animals receiving LJE, DUO, or LJE + DUO showed a decrease in cryptal damage and inflammation compared to the control group; LJE (300 mg/kg) and DUO + LJE (100 and 300 mg/kg) produced the highest protective effect. In addition, histological scoring revealed the possibility of synergy between LJE and DUO. DSS administration destroys the integrity of the mucosal barrier leading to the disruption of intestinal epithelial layer, mucosal and submucosal ulceration, and infiltration of inflammatory cells [30]. Thus, the protective effect of LJE, DUO, and their combination consists in inhibition of the inflammation-induced damage in intestinal tissues.

The clinical score is considered to reflect the state of gastrointestinal diseases and is used as a reliable assessment tool for IBD patients [18, 31]. DSS-induced colitis in mice presents a variety of clinical symptoms such as diarrhea, bloody stool, or behavioral changes [30]. In this study, LJE or DUO did not improve the clinical score (spontaneous behavior and posture, coat and piloerection, and cleaning of perianal lesion), which might be due to the severity of damage in the intestinal mucosa and/or short period of LJE and DUO administration.

In the current study, LJE dose-dependently improved the macroscopic score, indicating the healing of gross structural damage in mice with colitis. Although the macroscopic score did not entirely corresponded to the results of histological findings, the dose-dependent improvement of macroscopic signs could indicate the possibility of significant effects in the long-term colitis model. Several studies have shown that the macroscopic score could be a useful tool to assess the induction of inflammatory lesions and the severity of histological damage [21, 32], including intestinal edema and cecal thickness [28].

Weight loss is regarded as one of the major systemic symptoms of the colonic structural damage [30, 33], and previous studies showed that mice treated with DSS for 7 days were rapidly losing weight starting from day 4 [34, 35]. The present results showed a similar pattern of weight loss in the DSS group; however, LJE, DUO, or LJE + DUO did not protect mice against weight loss, possibly because of the severity of DSS-induced intestinal damage.

We have investigated the production of cytokines including IFN-*γ*, IL-1*β*, IL-6, IL-10, IL-12 (P40), IL-12 (P70), IL-17, and TNF-*α* in colonic mucosa of mice with experimental colitis. The levels of IL-1*β*, IL-6, and TNF-*α* in mice treated with LJE (300 mg/kg) and DUO + LJE (300 mg/kg) were significantly improved compared to those in other groups. The combined treatment with DUO + LJE inhibited DSS-induced changes in IL-1*β*, IL-6, and IL-12 (P40) compared to LJE or DUO alone. However, the levels of IFN-*γ* and IL-10 did not change compared to control. DSS damages the colonic epithelial barrier resulting in subsequent inflammation and induction of cytokine dysregulation, which is thought to be due to the imbalance of T helper (Th) cell subsets, Th1 and Th2 [36]. IBD is characterized by upregulation of Th1 cytokines including IFN-*γ*, IL-12 (P40), IL-12 (P70), and TNF-*α* and Th2 cytokines IL-4 and IL-5. Recent studies have also shown that IBD is closely associated with the production of Th17 cytokines such as IL-17 [19, 37]. In an acute model of DSS-induced colitis, mice exhibited an increase in IL-12 and IL-17, suggesting an association with Th1/Th17-dependent mechanisms [19]. Our results show that LJE inhibited IL-17, a representative Th17 cytokine, and Th1/Th17 response-related proinflammatory cytokines TNF-*α*, IL-1*β*, IL-6, and IL-12 (P40), which is consistent with previous results obtained with* Lonicera japonica *[38]. IL-10 contributes to the differentiation of regulatory T cells (T_reg_), while suppressing dendritic cell-associated Th1 and Th17 immunity [39]. IL-10 and IFN-*γ*, which showed no significant change in our experiments, were not elevated in acute and subacute stages of DSS-induced colitis [19].

At the same time, DUO alone also showed an anti-inflammatory effect by regulating IL-6, IL-12 (40), and IL-17. LAB such as* L. rhamnosus* and probiotic mixture composed of* L. acidophilus, B. lactis, L. plantarum, *and* B. breve* have shown protection against DSS-induced colitis [40, 41]. IL-10 was not elevated in our experiments, but several studies have reported that* Lactobacillus*,* Bifidobacterium* and* Streptococcus* species induce the production of anti-inflammatory IL-10, although with different effectiveness [42–44]. Thus,* Bifidobacterium* has been implicated in promoting T_reg_ differentiation and programming T_reg_ cells [45]. In our experiments, the measurements were performed only in intestinal mucosa at day 7 of DSS induction, when the differentiation or movement of T_reg_ cells might not have been completed and T_reg_ cells might still reside in submucosal layers. Plus, the level of IL-10 in mesenteric lymph node or spleen where most T_reg_ cells secreting IL-10 exist was not observed. Therefore, our results do not rule out the possibility that DUO could increase IL-10 levels, which should be evaluated in each step, region, and immunological tissue. DUO also might regulate Th17 and Th1/Th17 response-related cytokines. LJE and DUO showed a synergistic dose-dependent effect on IL-17, IL-1*β*, IL-6, and IL-12 (P40) production, indicating that LJE + DUO might activate the Th17- and Th1/Th17-dependent pathways similar to LJE and DUO alone. The effect of LJE and DUO on the level of Th17 or Th1/Th17 cytokines correlated with that on histopathology.

Taken together, LJE and DUO inhibited proinflammatory cytokines IL-1*β*, IFN-*γ*, TNF-*α*, IL-6, and IL-12 (P40), which are produced by activated macrophages or dendritic cells in mucosa inducing acute mucosal inflammation. These results correspond to the previous findings, suggesting that in the acute DSS-induced colitis the colonic Th cells exhibit Th17 and Th1/Th17 profiles [36]. The administration of LJE and DUO did not change the levels of IL-10 related to T_reg_ cells. Th1 cytokines have been shown to have a profound influence on the severity of inflammation and infection in mice at the acute phase of DSS-induced colitis, while Th2 cytokines are involved in chronic intestinal inflammation [46].

LJE administration positively affected the colon length, microscopic findings, histological scoring, and proinflammatory cytokine profile, suggesting a mechanism underlying LJE protective effect against cryptal gland loss and epithelial damage. The synergetic effect of LJE and DUO was demonstrated by the histological scoring and the production of cytokines including IL-1*β*, IL-6, and IL-12 (P40). Therefore, the protective effect of DUO might be associated with Th1 and Th17 differentiation and its neutralization, leading to prevention of cryptal damage, infiltration of inflammatory cells, and ulceration of intestinal tissues.

In the present study, we selected LJE dosage based on standard doses used in humans. In mice, LJE at 300 mg/kg corresponds to 167 mg/kg in humans considering the rate of excretion (10-fold compared to human) and LJE yield (18.01%). As a result, 300 mg/kg of LJE in mice corresponds to approximately 8 g of LJ raw material in a 50 kg human; this concentration is similar to LJ dosage prescribed in clinics [47, 48]. Based on this data, we used LJE in a concentration range from 100 (low) to 300 (high) mg/kg. DUO was approved by the Korean Food and Drug Administration at a dosage of 500 mg twice a day (total 1,000 mg a day), which corresponds to 200 mg/kg of adult body weight (50 kg). Considering this data and mouse excretion rate, in our experiments mice received DUO at a fixed dose of 300 mg/kg, which is similar to a standard human dosage.

It is noteworthy that this study showed a dose-dependent effect of LJE on clinical and biochemical parameters of experimental colitis; in addition, synergetic effects with DUO were observed. Experiments with modified LJE dosage and long-term administration are needed in the future.

## 5. Conclusions

Our results demonstrated that LJE was effective in protection against colitis in mice and that cotreatment with LJE and DUO showed synergy in DSS-induced IBD model. The possibility of a synergetic effect of LJE and DUO cotreatment was strongly suggested. LJE at 300 mg/kg was the most effective dose for improvement of IBD symptoms and pathology in mice. LJE and DUO might have a beneficial effect on colitis by regulating Th17- or Th1/Th17-related immune mechanisms. This study presents an evidence of LJE protective effects on IBD, suggesting a possibility for a clinical trial. Further studies on the immune mechanisms induced by LJE should be based on a broader LJE dose range and long-term administration.
